# Acidosis inhibits rhythmic contractions of human thoracic ducts

**DOI:** 10.14814/phy2.14074

**Published:** 2019-04-25

**Authors:** Anders L. Moeller, Vibeke E. Hjortdal, Donna M. B. Boedtkjer, Ebbe Boedtkjer

**Affiliations:** ^1^ Department of Biomedicine Aarhus University Aarhus Denmark; ^2^ Department of Clinical Medicine Aarhus University Aarhus Denmark

**Keywords:** Acidosis, edema, lymphatics, thoracic duct

## Abstract

Lymph vessels counteract edema by transporting interstitial fluid from peripheral tissues to the large veins and serve as conduits for immune cells, cancer cells, and pathogens. Because edema during inflammation and malignancies is frequently associated with acidosis, we tested the hypothesis that acid‐base disturbances affect human thoracic duct contractions. We studied, by isometric and isobaric myography, the contractile function of human thoracic duct segments harvested with written informed consent from patients undergoing esophageal cancer surgery. Human thoracic ducts produce complex contractile patterns consisting of tonic rises in tension (isometric myography) or decreases in diameter (isobaric myography) with superimposed phasic contractions. Active tone development decreases substantially (~90% at 30 vs. 7 mmHg) at elevated transmural pressure. Acidosis inhibits spontaneous as well as noradrenaline‐ and serotonin‐induced phasic contractions of human thoracic ducts by 70–90% at extracellular pH 6.8 compared to 7.4 with less pronounced effects observed at pH 7.1. Mean tension responses to noradrenaline and serotonin – averaged over the entire period of agonist exposure – decrease by ~50% at extracellular pH 6.8. Elevating extracellular [K^+^] from the normal resting level around 4 mmol/L increases overall tension development but reduces phasic activity to a level that is no different between human thoracic duct segments investigated at normal and low extracellular pH. In conclusion, we show that extracellular acidosis inhibits human thoracic duct contractions with more pronounced effects on phasic than tonic contractions. We propose that reduced phasic activity of lymph vessels at low pH attenuates lymph propulsion and increases the risk of edema formation.

## Introduction

Peripheral edema – defined as tissue swelling due to interstitial fluid accumulation – is a frequent condition and significant complication of surgical interventions and commonly prescribed medications. At steady‐state, lymphatic drainage balances net fluid filtration, which is determined by the transmural hydrostatic and colloid osmotic pressure differences across the capillaries. Thus, edema can arise because of increased net fluid filtration from capillaries or reduced capacity for lymphatic drainage (Taylor 1981).

Acid‐base disorders have a multitude of pathogeneses: they can be systemic due to respiratory, renal, or metabolic dysfunction, or stem from local pathologies such as ischemia, inflammation, or cancer. Ischemic, inflamed, and malignant tissues are also often edematous. Whereas extracellular acidosis relaxes blood vessels (Boedtkjer 2018) and thereby reduces precapillary resistance, which elevates capillary hydrostatic pressure and filtration, effects of acid‐base disturbances on human lymph vessel contractions have not previously been defined. Edema in response to vasorelaxation is typically ascribed to changes in Starling forces in the capillaries but the relative importance of enhanced net fluid filtration versus inhibited lymph drainage is in many cases unclear.

From the lymphatic capillaries – that consist of only a single endothelial cell layer – the lymph collects in pre‐ and postnodal lymph vessels with a growing smooth muscle cell layer before it ends up in the larger lymph conduits including the thoracic duct (Margaris and Black 2012). This transport from the initial lymphatics toward the great veins typically moves the lymph against a steady‐state pressure gradient and gravitational forces. Extrinsic and intrinsic pumping activity along the entire lymphatic network is required for lymph transport and hence for the lymphatic system to perform its homeostatic functions (Zawieja 2009). Lymph vessels consist of a long series of lymphangions, each formed by a lymph vessel segment demarcated in both ends by unidirectional valves (Mislin 1961); and the continuous propulsion of lymph by the thoracic duct reduces the afterload that must be overcome in order for more peripheral lymph vessels to move lymph toward the inlet in the large veins.

The wall of the human thoracic duct is composed of a luminal endothelial cell layer, underlying interstitial Cajal‐like cells, and a variable media that contains strays of vascular smooth muscle cell bundles running circumferentially, obliquely, and longitudinally relative to the direction of the lymph vessel (Briggs Boedtkjer et al. 2013). Coordinated contractions and relaxations of neighboring lymphangions create the local transitory pressure differences that propel lymph from one lymphangion to the next from the peripheral tissues in direction of the central inlet into the subclavian vein. Consistent with this role for lymph pumping, spontaneous human thoracic duct contractions and responses to agonist stimulation are typically rhythmic and phasic although more tonic contractions can occur in response to high agonist concentrations (Sjoberg and Steen 1991b; Telinius et al. 2010).

Measurements of steady‐state pH of thoracic duct lymph vary substantially between individuals but at least in dogs are generally between 7.0 and 7.5 (Carlsten and Söderholm 1960; Witte et al. 1967; Berman et al. 1969; Takaori and Tosaki 1978; Palmer et al. 1998). Severe acidification of thoracic duct lymph occurs during hemorrhagic and endotoxin shock (Berman et al. 1969); and systemic acid loading of dogs reduces pH of blood and thoracic duct lymph to comparable extents (Witte et al. 1967). Locally, the acid‐base conditions of lymph reflects interstitial levels in the drained organ (Palmer et al. 1998).

In the current study, we tested the hypothesis that acidosis inhibits the magnitude and oscillatory pattern of thoracic duct contractions. We show that moderate decreases in extracellular pH (pH_o_ 7.1) can inhibit phasic contractions of thoracic ducts whereas severe decreases in pH (pH_o_ 6.8) reduce the overall tension development and almost completely abolish contractile rhythmicity. We propose that attenuated lymph vessel contractions during acidosis can contribute to local accumulation of interstitial fluid and manifest as edema.

## Materials and Methods

We received segments of human thoracic ducts from esophageal cancer resections at Aarhus University Hospital, Denmark. The Regional Committee on Health Research Ethics (M‐20070194) and the Danish Data Protection Agency (2007‐57‐0010) approved the procedure for obtaining human thoracic ducts. The study included 18 male and six female patients (average age: 68 ± 8 (SD) years), who gave written informed consent and underwent uncomplicated surgery.

### Isometric myography

Human thoracic ducts were cut into 2 mm long ring segments that were mounted on 40‐*μ*m stainless steel wires in 4‐channel myographs (610M; DMT, Denmark). The relaxed lymphatic vessels were normalized to an internal diameter equivalent to a transmural pressure of 20.6 mmHg based on LaPlace's law (Mulvany and Halpern 1977; Telinius et al. 2010). Following normalization, the human thoracic duct segments recovered in physiological saline solution (see composition below) aerated with 5% CO_2_/balance air at pH 7.4 for approximately 45 min at 37°C. Viability was confirmed by contractile response to 10 *μ*mol/L noradrenaline. Individual thoracic duct segments were randomly assigned to investigation under one of the following test conditions: (a) time control, pH 7.4, 5% CO_2_, 22 mmol/L HCO_3_^–^, (b) respiratory acidosis (RAc), pH 7.1, 10% CO_2_, 22 mmol/L HCO_3_^–^, (c) moderate metabolic acidosis (MAc), pH 7.1, 5% CO_2_, 11 mmol/L HCO_3_^–^, or (d) severe metabolic acidosis (MAc), pH 6.8, 5% CO_2_, 5.5 mmol/L HCO_3_^–^. Contractions to cumulative increases of [serotonin] from 10 nmol/L to 30 *μ*mol/L in half‐log steps, [noradrenaline] from 10 nmol/L to 30 *μ*mol/L in half‐log steps, and [K^+^] from 4 mmol/L to 120 mmol/L were now evaluated in each vessel under the assigned acid‐base condition and under control conditions (pH 7.4, 5% CO_2_, 22 mmol/L HCO_3_^–^). When the buffer composition was changed from the assigned test condition to the control condition, or *vice versa*, we waited for 25 min in order to reach a new steady‐state. We constructed cumulative concentration‐response curves to noradrenaline, serotonin, and extracellular K^+^ by exposing thoracic duct segments to each concentration step for 3 min before addition of the next concentration or washout. Concentration‐response relationships for extracellular K^+^ were established in the presence of 1–2 *μ*mol/L of the *α*‐receptor blocker phentolamine in order to avoid effects of noradrenaline released from perivascular nerves in response to depolarization (Telinius et al. 2014a). We performed the time control experiments on matched thoracic duct segments investigated in parallel with – and thus experiencing similar time delays as – the segments exposed to acidosis. At the end of the experiments, force development of all thoracic duct segments was tested in response to 120 mmol/L extracellular K^+^ under control conditions (pH 7.4, 5% CO_2_, 22 mmol/L HCO_3_^–^), and all contractile responses from that thoracic duct segment were normalized to this standardized force response. The normalization procedure was performed in order to minimize variation caused by differences in maximal contractile capacity between the lymphatic segments, for instance, due to uneven content of longitudinally and circumferentially orientated smooth muscle cell bundles (Briggs Boedtkjer et al. 2013).

### Isobaric myography

Human thoracic duct segments (>4 mm long) were mounted with silk sutures on plastic cannulas in pressure myographs (DMT) and tested for myogenic tone development and contraction in response to noradrenaline at transmural pressures of 7 and 20 mmHg. The transmural pressures (in mmHg) are given as whole integers because they – due to small variations in tissue positioning and intravascular pressure control – can vary slightly between experiments. Movies of the pressurized human thoracic ducts were recorded with a USB microscope camera (The Imaging Source, Germany) and analyzed with MyoView 3.2 (DMT). At the end of the experiment, the fully relaxed diameter was recorded by exposing the thoracic duct segments to Ca^2+^‐free salt solution and 10 *μ*mol/L of the phosphodiesterase inhibitor papaverine.

Active tone development in pressure myograph experiments was calculated based on the formula: Active Tone* *= (*D*_passive_ – *D*_active_)/*D*_passive_, where *D*_active_ is the outer diameter under the given condition and *D*_passive_ is the outer diameter observed at the same transmural pressure when the thoracic duct segment was exposed to Ca^2+^‐free salt solution with 10 *μ*mol/L papaverine. We used outer diameter for these calculations because the inner diameter is poorly defined in the image sequences due to the substantial thickness of the human thoracic duct wall reducing optical transparency.

### Quantification of phasic contractile activity

**Figure 1:**
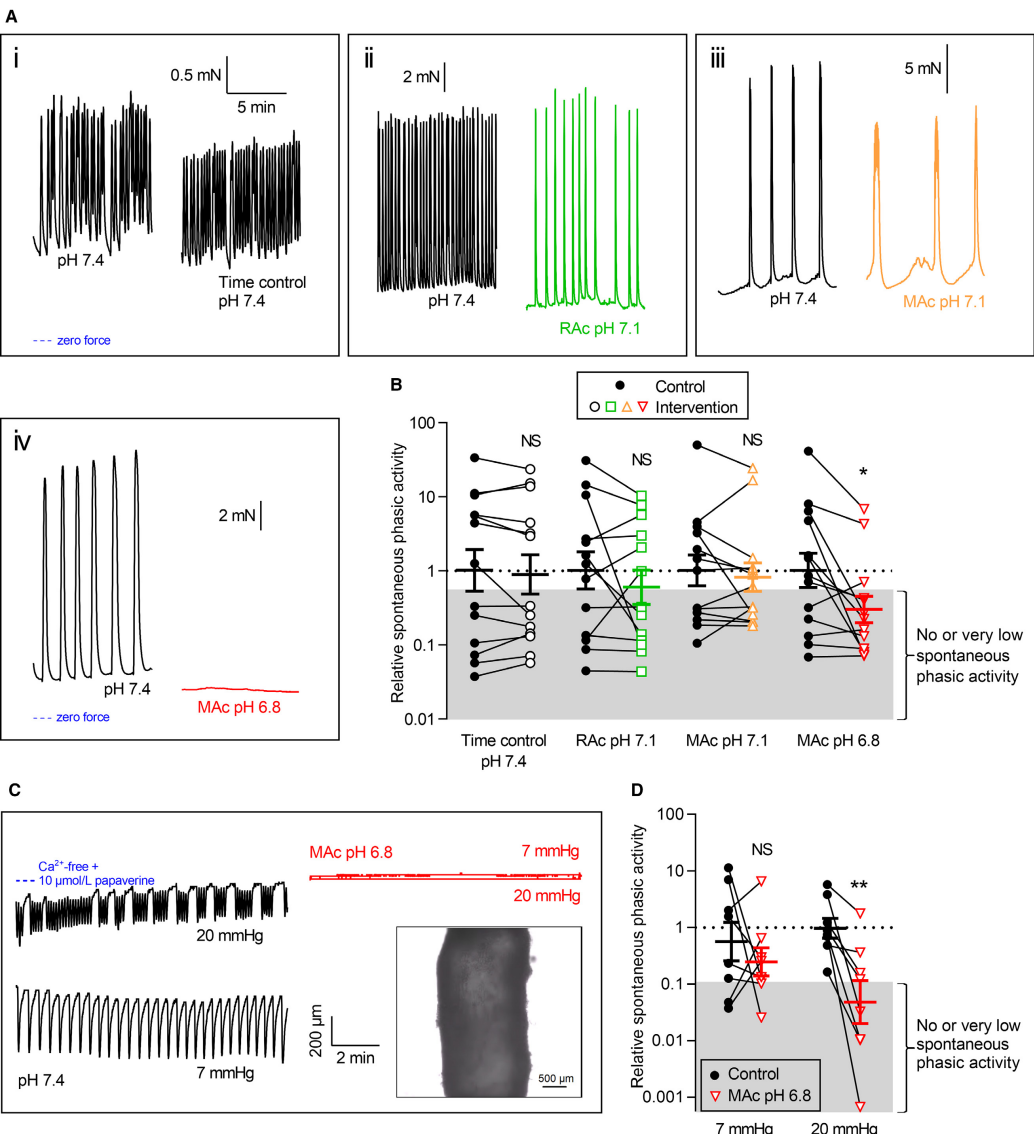
Extracellular acidosis inhibits spontaneous contractions of human thoracic ducts. (A+B) Original traces from individual experiments (A) and average data (B) showing spontaneous phasic contractions of human thoracic duct segments mounted under isometric conditions (*n* = 13). In panel A, all four sets of traces are plotted on the time scale shown in the first subpanel. The first trace in each subpanel of panel A represents control extracellular conditions (pH 7.4, 5% CO_2_ and 22 mmol/L HCO_3_^–^) whereas the second trace represents the respective intervention: time control (*i*, unchanged acid‐base conditions), respiratory acidosis (*ii*, pH reduced to 7.1 by increasing CO_2_ to 10%), and metabolic acidosis (pH reduced to, *iii*, 7.1 or, *iv*, 6.8 by lowering [HCO_3_^–^] to 11 or 5.5 mmol/L). We performed the time control experiments on matched thoracic duct segments investigated in parallel with – and thus experiencing similar time delays as – the segments exposed to acidosis. The phasic contractile activity – calculated as the average numerical value of the first derivative of the tension curve under each experimental condition – represents a combined measure of amplitude and frequency, which we express relative to the matched average value at pH 7.4. The force axes are aligned in each row of panel A and the zero level indicated by the blue horizontal dotted lines. In panel B, symbols that fall within the gray area correspond to lymphatic vessels showing minimal, if any, spontaneous phasic activity as illustrated by the red trace in panel A, *iv*. (C+D) Original traces (C) and average data (D) showing spontaneous contractions of human thoracic duct segments mounted under isobaric conditions (*n* = 8). Insert shows an exemplar image of a human thoracic duct segment during isobaric investigation. The two traces on the left represent control extracellular conditions (pH 7.4, 5% CO_2_ and 22 mmol/L HCO_3_^–^) whereas the two traces on the right represent metabolic acidosis of pH 6.8 (obtained by reducing [HCO_3_^–^] to 5.5 mmol/L). The phasic contractile activity is expressed relative to the average value of thoracic ducts investigated at a transmural pressure of 20 mmHg at pH 7.4. The fully relaxed diameter – measured under Ca^2+^‐free conditions with 10 *μ*mol/L papaverine – is essentially identical at 7 and 20 mmHg and indicated by the blue horizontal dotted line in panel C. In panel D, symbols that fall within the gray area correspond to lymphatic vessels showing minimal, if any, spontaneous phasic activity as illustrated by the red traces in panel C. We compared data by two‐tailed Student's *t*‐tests. *P <* *0.05, **P <* *0.01, NS: not significantly different versus control at pH_o_ 7.4. RAc: respiratory acidosis, MAc: metabolic acidosis.

The contractile patterns differed substantially between individual thoracic duct segments from regular monophasic contractions to complex patterns with secondary spikes on top of large primary contractions. Variation is typical for spontaneous and agonist‐induced rhythmic contractions of lymph vessels (McHale and Meharg 1992; Telinius et al. 2010). To provide a simple, quantitative measure of overall phasic contractile activity, we calculated the average numerical value of the first derivative of the tension (isometric myography) and diameter (isobaric myography) curves under each experimental condition. This approach quantifies the oscillatory activity and although it does not specifically reveal whether the amplitude and/or frequency is affected, it is much less sensitive to interindividual variation and therefore more robust in human patient populations that are inherently genetically and phenotypically diverse. In order to ease the interpretation of this derived ”phasic activity” parameter, the gray areas in Figure 1B and D illustrate intervals where no obvious phasic activity – beyond that which could be explained by noise – was apparent by visual inspection of the original traces. The applied statistical analyses, however, do not rely on this subjective, arbitrary separation between vessels that show phasic activity and those that do not. Instead, they include the complete data sets in order to eliminate the risk of selection bias.

### Solutions

The standard physiological salt solution used for functional experiments contained (in mmol/L): 140 Na^+^, 4 K^+^, 1.6 Ca^2+^, 1.2 Mg^2+^, 124 Cl^–^, 22 HCO_3_^–^, 1.2 SO_4_^2–^, 1.2 H_2_PO_4_^–^, 10 HEPES, 5.5 glucose, and 0.03 ethylenediamine‐tetraacetic acid (EDTA). After bubbling with a gas mixture of 5% CO_2_/balance air, pH was adjusted to 7.4. Salt solutions mimicking metabolic acidosis were obtained by substitution of HCO_3_^–^ with equimolar Cl^–^ and were adjusted to pH 7.1 (11 mmol/L HCO_3_^–^) or 6.8 (5.5 mmol/L HCO_3_^–^) when aerated with 5% CO_2_/balance air. Salt solutions mimicking respiratory acidosis were obtained by bubbling with a gas mixture of 10% CO_2_/balance air and pH adjusted to 7.1. Solutions with elevated [K^+^] were obtained by equimolar substitution for Na^+^. Ca^2+^‐free solutions were without the 1.6 mmol/L Ca^2+^ and associated 3.2 mmol/L Cl^–^ and any remaining free Ca^2+^ was buffered by addition of 5 mmol/L ethylene glycol‐bis(*β*‐aminoethyl ether)‐*N*,*N*,*N*’,*N*’‐tetraacetic acid (EGTA).

### Statistics

Data are expressed as mean ± SEM unless otherwise specified. For each analysis, *n* equals number of patients; if more than one thoracic duct segment was investigated from the same patient under any given condition, the average of those measurements was used to represent that patient. *P *<* *0.05 was considered statistically significant. Statistical analyses performed using GraphPad Prism 7.03 software are detailed for each comparison in the text or corresponding figure legend. We addressed potential effects of time by alternating the order of interventions and/or by performing parallel time control experiments.

## Results

We studied contractile patterns of isolated human thoracic duct segments under control conditions and at different levels of extracellular acidification. Metabolic acidosis was mimicked by reducing pH_o_ and [HCO_3_^–^]_o_ at constant pCO_2_, whereas respiratory acidosis was mimicked by reducing pH_o_ and elevating pCO_2_ at constant [HCO_3_^–^]_o_.

### Extracellular acidosis inhibits spontaneous contractions of human thoracic ducts

The passive internal diameter of human thoracic duct segments was 1794 ± 145 *μ*m (*n* = 13) when mounted in wire myographs and normalized to an equivalent transmural pressure of 20.6 mmHg. Around 60% of the investigated human thoracic duct segments produced robust spontaneous phasic contractions when mounted in wire myographs under control conditions at pH_o_ 7.4 (Fig. 1A,B; symbols that fall within the gray area correspond to thoracic duct segments showing minimal, if any, spontaneous phasic activity). After mounting and normalization, the thoracic duct segments rested for approximately 45 min before they were exposed to agonists. The occurrence of spontaneous contractions typically increased during this prolonged incubation period. Figure 1A shows examples of original force traces. As illustrated, the rhythmic contractile patterns varied substantially with some thoracic duct segments showing simple and others more composite oscillatory contractile configurations. Irrespectively, extracellular acidification blunted the spontaneous rhythmic contractile responses of human thoracic duct segments when pH_o_ from its control level of 7.4 was decreased to 6.8, which reduced oscillations by approximately 70% (Fig. 1A and B; note the log‐scale).

Mounted in pressure myographs, human thoracic duct segments (*n* = 8) – that were fully relaxed by exposure to Ca^2+^‐free bath solution containing 10 *μ*mol/L papaverine – had external diameter of 2546 ± 134 *μ*m at a transmural pressure of 20 mmHg. All the human thoracic duct segments investigated under isobaric conditions at pH_o_ 7.4 showed spontaneous oscillations in lumen diameter at transmural pressures of either 7 or 20 mmHg (Fig. 1C; symbols that fall within the gray area correspond to thoracic duct segments showing minimal, if any, spontaneous phasic activity). At a transmural pressure of 20 mmHg, extracellular metabolic acidosis of pH_o_ 6.8 inhibited the phasic contractile activity by more than 90% (Fig. 1C and D; note the log‐scale).

### Extracellular acidosis inhibits noradrenaline‐ and serotonin‐induced contractions of isometrically tested human thoracic ducts

**Figure 2:**
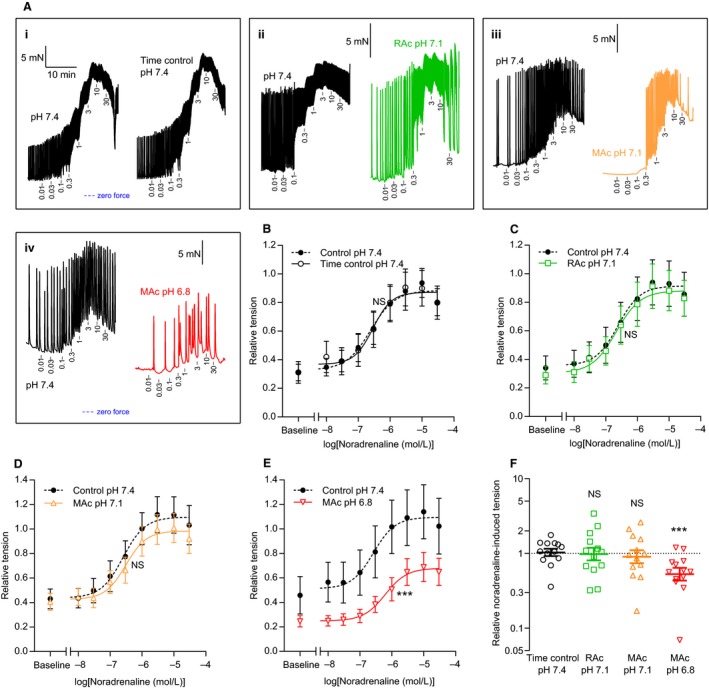
Extracellular acidosis inhibits overall noradrenaline‐induced contractions of human thoracic ducts. (A–E) Original traces (A) and average data (B–E) showing noradrenaline‐induced contractions of human thoracic duct segments mounted under isometric conditions (*n* = 13). The concentrations of noradrenaline present after each cumulative application are indicated in *μ*mol/L on the traces. In panel A, all four sets of traces are plotted on the time scale shown in the first subpanel. The first trace in each subpanel of panel A represents control extracellular conditions (pH 7.4, 5% CO_2_ and 22 mmol/L HCO_3_^–^) whereas the second trace represents the respective intervention: time control (*i*, unchanged acid‐base conditions), respiratory acidosis (*ii*, pH reduced to 7.1 by increasing CO_2_ to 10%), and metabolic acidosis (pH reduced to, *iii*, 7.1 or, *iv*, 6.8 by lowering [HCO_3_^–^] to 11 or 5.5 mmol/L). We performed the time control experiments on matched thoracic duct segments investigated in parallel with – and thus experiencing similar time delays as – the segments exposed to acidosis. The force axes are aligned in each row of panel A and the zero level indicated by the blue horizontal dotted lines. The tension values in panel B through E are expressed relative to that developed during exposure to 120 mmol/L extracellular K^+^ under control conditions (pH 7.4, 5% CO_2_, 22 mmol/L HCO_3_^–^) at the end of each experiment. (F) Noradrenaline‐induced cumulative contractile responses calculated as the area between the curves and the matched baseline values in panel B through E (*n* = 13). Note that the data are expressed relative to the matched control curve at pH 7.4 and plotted on a log‐scale. We compared data in panel B‐E by extra sum‐of‐squares *F*‐tests (results are shown in Table 1) and in panel F by repeated measures one‐way ANOVA followed by Dunnett's posttest. ***P <* *0.001, NS: not significantly different versus time control at pH_o_ 7.4. RAc: respiratory acidosis, MAc: metabolic acidosis.

**Figure 3:**
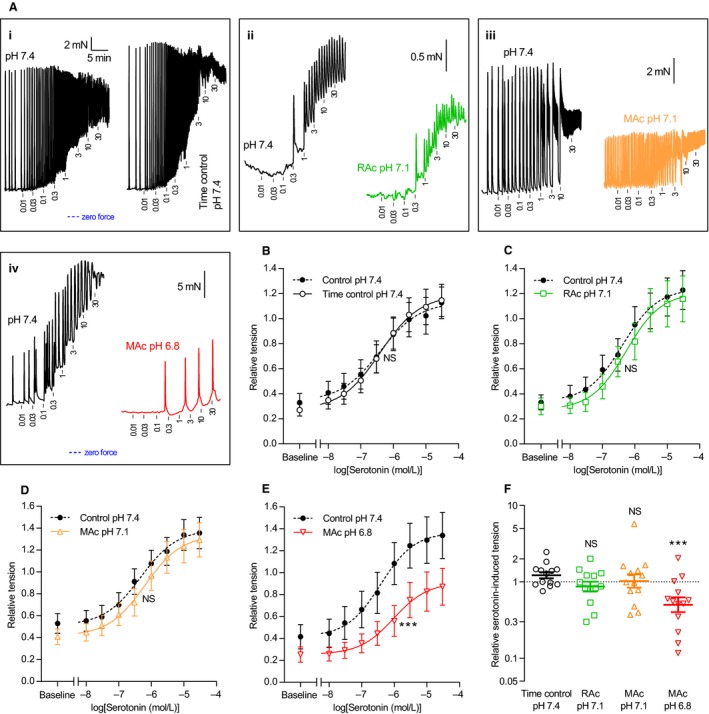
Extracellular acidosis inhibits overall serotonin‐induced contractions of human thoracic ducts. (A–E) Original traces (A) and average data (B–E) showing serotonin‐induced contractions of human thoracic duct segments mounted under isometric conditions (*n* = 13). The concentrations of serotonin present after each cumulative application are indicated in *μ*mol/L on the traces. In panel A, all four sets of traces are plotted on the time scale shown in the first subpanel. The first trace in each subpanel of panel A represents control extracellular conditions (pH 7.4, 5% CO_2_ and 22 mmol/L HCO_3_^–^) whereas the second trace represents the respective intervention: time control (*i*, unchanged acid‐base conditions), respiratory acidosis (*ii*, pH reduced to 7.1 by increasing CO_2_ to 10%), and metabolic acidosis (pH reduced to, *iii*, 7.1 or, *iv*, 6.8 by lowering [HCO_3_^–^] to 11 or 5.5 mmol/L). We performed the time control experiments on matched thoracic duct segments investigated in parallel with – and thus experiencing similar time delays as – the segments exposed to acidosis. The force axes are aligned in each row of panel A and the zero level indicated by the blue horizontal dotted lines. The tension values in panel B through E are expressed relative to that developed during exposure to 120 mmol/L extracellular K^+^ under control conditions (pH 7.4, 5% CO_2_, 22 mmol/L HCO_3_^–^) at the end of each experiment. (F) Serotonin‐induced cumulative contractile responses calculated as the area between the curves and the matched baseline value in panel B through E (*n* = 13). Note that the data are expressed relative to the matched control curve at pH 7.4 and plotted on a log‐scale. We compared data in panel B‐E by extra sum‐of‐squares *F*‐tests (results are shown in Table 2) and in panel F by repeated measures one‐way ANOVA followed by Dunnett's posttest. ***P <* *0.001, NS: not significantly different versus time control at pH_o_ 7.4. RAc: respiratory acidosis, MAc: metabolic acidosis.

Application of noradrenaline or serotonin increased tone development in isometrically mounted human thoracic duct segments (Figs. 2 and 3). Tension development rose concentration‐dependently and reached a plateau at high agonist concentrations. Agonist‐induced mean tension development (i.e., the increase from baseline at the given pH_o_) was unaffected by moderate metabolic or respiratory acidosis of pH_o_ 7.1 (Figs. 2C,D,F and 3C,D,F) but almost 50% reduced when pH_o_ was lowered to 6.8 (Figs. 2E,F and 3E,F). Statistical parameters are provided in Tables 1 and 2.

**Table 1:** Concentration‐dependent contractions of human thoracic ducts in response to noradrenaline are inhibited at low pH. The displayed parameters – corresponding to Figure 2B through E – result from least‐squares fits to sigmoidal functions and were compared between acidosis (or time control) and the matched internal control based on extra sum‐of‐squares *F*‐tests. We performed the time control experiments on matched thoracic duct segments investigated in parallel with – and thus experiencing similar time delays as – the segments exposed to acidosis. Tension values are expressed relative to the tension development in response to 120 mmol/L extracellular K^+^ under control conditions at the end of each experiment

	Bottom: fitted resting tension	Top: fitted maximum tension	log(EC_50_ (mol/L))	
Control pH 7.4	0.359 ± 0.069	0.878 ± 0.068	–6.55 ± 0.28	
Time control pH 7.4	0.337 ± 0.071	0.878 ± 0.067	–6.60 ± 0.26	*P *=* *0.996
Control pH 7.4	0.354 ± 0.086	0.917 ± 0.081	–6.62 ± 0.32	
RAc pH 7.1	0.309 ± 0.088	0.879 ± 0.080	–6.66 ± 0.31	*P *=* *0.943
Control pH 7.4	0.440 ± 0.076	1.096 ± 0.072	–6.60 ± 0.23	
MAc pH 7.1	0.424 ± 0.072	0.986 ± 0.073	–6.51 ± 0.27	*P *=* *0.458
Control pH 7.4	0.512 ± 0.101	1.096 ± 0.098	–6.58 ± 0.35	
MAc pH 6.8	0.249 ± 0.085	0.679 ± 0.110	–6.21 ± 0.48	*P *<* *0.001

RAc, respiratory acidosis; MAc, metabolic acidosis.

*P*‐values refers to the comparisons between acidosis (or time control) and the matched internal control reported in the row above.

**Table 2:** Concentration‐dependent contractions of human thoracic ducts in response to serotonin are inhibited at low pH. The displayed parameters – corresponding to Figure 3B through E – result from least‐squares fits to sigmoidal functions and were compared between acidosis (or time control) and the matched internal control based on extra sum‐of‐squares *F*‐tests. We performed the time control experiments on matched thoracic duct segments investigated in parallel with – and thus experiencing similar time delays as – the segments exposed to acidosis. Tension values are expressed relative to the tension development in response to 120 mmol/L extracellular K^+^ under control conditions at the end of each experiment

	Bottom: fitted resting tension	Top: fitted maximum tension	log(EC_50_ (mol/L))	
Control pH 7.4	0.341 ± 0.092	1.132 ± 0.120	–6.42 ± 0.36	
Time control pH 7.4	0.273 ± 0.094	1.189 ± 0.129	–6.40 ± 0.32	*P *=* *0.867
Control pH 7.4	0.341 ± 0.097	1.239 ± 0.125	–6.38 ± 0.31	
RAc pH 7.1	0.275 ± 0.094	1.207 ± 0.136	–6.27 ± 0.31	*P *=* *0.666
Control pH 7.4	0.513 ± 0.091	1.391 ± 0.126	–6.33 ± 0.32	
MAc pH 7.1	0.417 ± 0.088	1.338 ± 0.140	–6.20 ± 0.31	*P *=* *0.323
Control pH 7.4	0.417 ± 0.120	1.367 ± 0.150	–6.42 ± 0.34	
MAc pH 6.8	0.255 ± 0.096	0.910 ± 0.170	–6.02 ± 0.52	*P *<* *0.001

RAc, respiratory acidosis; MAc, metabolic acidosis.

*P*‐values refers to the comparisons between acidosis (or time control) and the matched internal control reported in the row above.

### Extracellular acidosis inhibits noradrenaline‐ and serotonin‐induced oscillatory contractions of isometrically tested human thoracic ducts

**Figure 4:**
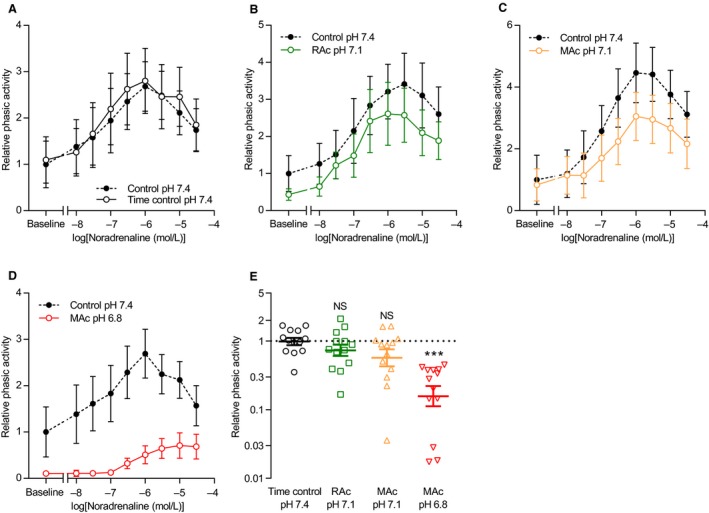
Extracellular acidosis inhibits phasic contractile activity of human thoracic ducts during noradrenaline stimulation. (A–D) Phasic contractile activity of human thoracic duct segments mounted under isometric conditions and stimulated with noradrenaline (*n* = 13) is expressed relative to the baseline value under control conditions (pH 7.4, 5% CO_2_, 22 mmol/L HCO_3_^–^). Corresponding original traces are shown in Figure 2A. We performed the time control experiments on matched thoracic duct segments investigated in parallel with – and thus experiencing similar time delays as – the segments exposed to acidosis. The phasic contractile activity – calculated as the average numerical value of the first derivative of the tension curve under each experimental condition – represents a combined measure of amplitude and frequency. (E) Cumulative phasic activity calculated as the area under the curves in panels A through D (*n* = 13). Note that the data are expressed relative to the matched control curve at pH 7.4, plotted on a log‐scale, and compared by repeated measures one‐way ANOVA followed by Dunnett's posttest. ***P <* *0.001, NS: not significantly different versus time control at pH_o_ 7.4. RAc: respiratory acidosis, MAc: metabolic acidosis.

**Figure 5:**
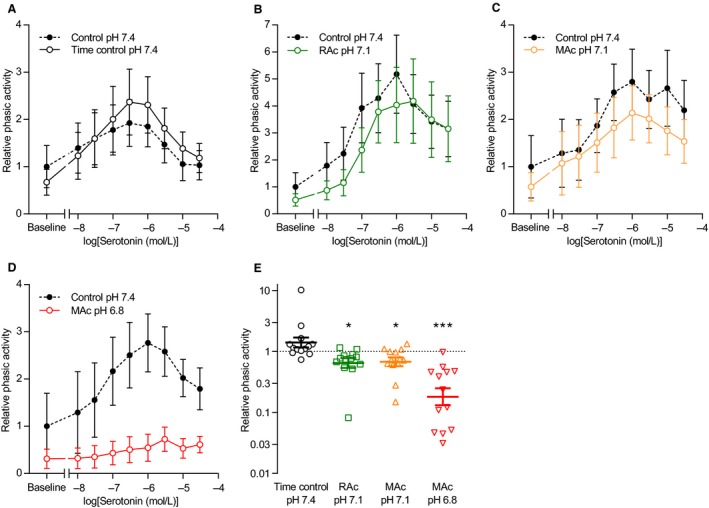
Extracellular acidosis inhibits phasic contractile activity of human thoracic ducts during serotonin stimulation. (A–D) Phasic activity of human thoracic duct segments mounted under isometric conditions and stimulated with serotonin (*n* = 13) is expressed relative to the baseline value under control conditions (pH 7.4, 5% CO_2_, 22 mmol/L HCO_3_^–^). Corresponding original traces are shown in Figure 3A. We performed the time control experiments on matched thoracic duct segments investigated in parallel with – and thus experiencing similar time delays as – the segments exposed to acidosis. The phasic contractile activity – calculated as the average numerical value of the first derivative of the tension curve under each experimental condition – represents a combined measure of amplitude and frequency. (E) Cumulative phasic activity calculated as the areas under the concentration‐response curves of panel A through E (*n* = 13). Note that the data are expressed relative to the matched control curve at pH 7.4, plotted on a log‐scale, and compared by repeated measures one‐way ANOVA followed by Dunnett's posttest. ***P <* *0.001, NS: not significantly different versus time control at pH_o_ 7.4. RAc: respiratory acidosis, MAc: metabolic acidosis.

Agonist stimulation induced rhythmic phasic contractions that occurred also in human thoracic duct segments that displayed no or very limited spontaneous phasic activity in the absence of agonists (Figs. 2A and 3A). The relationship between the concentration of noradrenaline or serotonin and the oscillatory behavior of the human thoracic duct was bell‐shaped with maximal phasic activity observed at concentrations close to 1 *μ*mol/L (Figs. 4 and 5). Extracellular acidosis inhibited the induced phasic contractions across the whole range of tested agonist concentrations (Figs. 4 and 5). For noradrenaline, the inhibitory effect of acidosis on phasic activity became significant only when pH_o_ was lowered to 6.8 and under these conditions amounted to more than 80% (Fig. 4E). For serotonin, the inhibitory effect of acidosis on rhythmic contractions was significant and at a magnitude of approximately 30% when pH_o_ was decreased from 7.4 to 7.1 and around 80% when pH_o_ was decreased to 6.8 (Fig. 5E).

### Extracellular acidosis inhibits stretch‐ and noradrenaline‐induced active tone in isobarically tested human thoracic ducts

**Figure 6:**
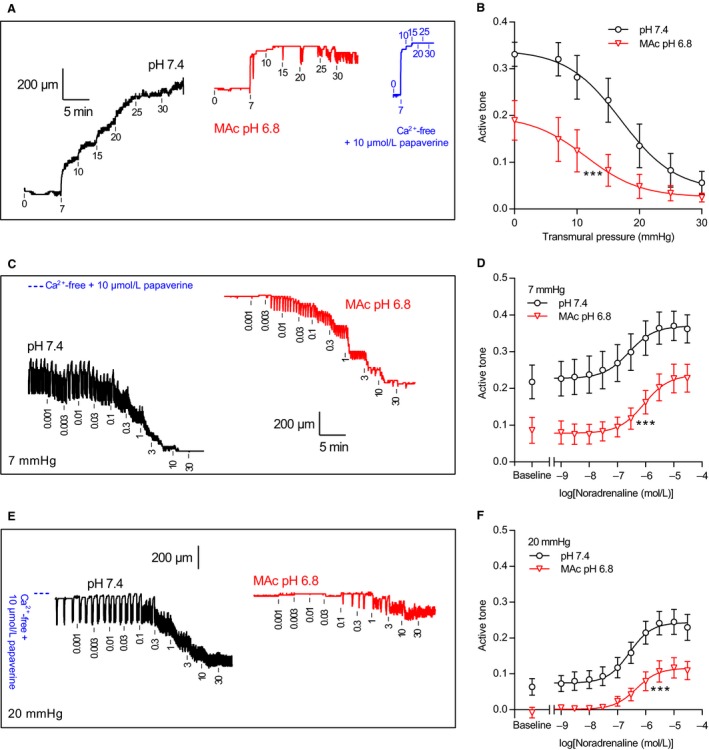
Extracellular acidosis inhibits myogenic tone and noradrenaline‐induced contractions of human thoracic ducts under isobaric conditions. (A+B) Original traces (A) and average data (B, *n* = 8) showing the response to transmural pressure changes of human thoracic ducts mounted under isobaric conditions. The transmural pressures are indicated on the traces in mmHg. (C–F) Original traces (C+E) and average data (D+F, *n* = 8) showing noradrenaline‐induced contractions of human thoracic duct segments mounted under isobaric conditions at transmural pressures of 7 mmHg (C+D) or 20 mmHg (E+F). The noradrenaline concentrations are indicated on the traces in *μ*mol/L. The traces in panel C and E are plotted on the same time scale shown in panel C. The blue lines in panels A, C, and E indicate the fully relaxed diameters achieved under Ca^2+^‐free conditions with 10 *μ*mol/L papaverine. Curves in panel B, D, and F are the results of least‐squares fits to sigmoidal functions and were compared by extra sum‐of‐squares *F*‐tests (results are shown in Table 3). ***P <* *0.001 versus pH_o_ 7.4. MAc: metabolic acidosis.

**Figure 7:**
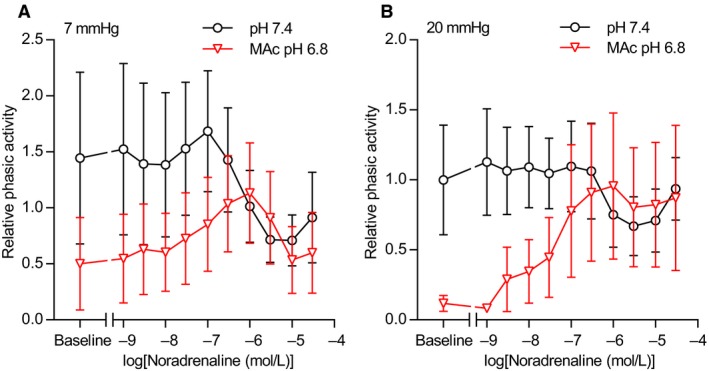
Noradrenaline and extracellular pH modify phasic activity of human thoracic ducts mounted under isobaric conditions. (A+B) Phasic activity of human thoracic duct segments mounted under isobaric conditions at transmural pressures of 7 mmHg (A) or 20 mmHg (B) and stimulated with noradrenaline (*n* = 8). In both panels, the phasic activity – calculated as the average numerical value of the first derivative of the diameter curve under each experimental condition – represents a combined measure of amplitude and frequency, which we express relative to the baseline value at pH 7.4 and a transmural pressure of 20 mmHg. Corresponding original traces are shown in Figure 6C and E. MAc: metabolic acidosis.

The human thoracic duct segments mounted under isobaric conditions showed greater active tone development under low compared to high transmural pressures (Figs. 6A,B and 7). This finding is consistent with previous results from rat lymphatic vessels (Mizuno et al. 1997). When pH_o_ was reduced from 7.4 to 6.8, overall active tone development across the tested range of transmural pressures between 0 and 30 mmHg was significantly attenuated (Fig. 6A and B and Table 3).

**Table 3:** Constrictions of human thoracic ducts in response to transmural pressure or noradrenaline stimulation are inhibited at low pH. The displayed parameters – corresponding to Figure 6B, D and F – result from least‐squares fits to sigmoidal functions and were compared between acidosis and the matched internal control based on extra sum‐of‐squares *F*‐tests

	Bottom: fitted minimum active tone	Top: fitted maximum active tone	log(EC_50_ (mmHg or mol/L))	
Transmural pressure (Fig. 6B)				
Control pH 7.4	0.040 ± 0.053	0.340 ± 0.040	17.07 ± 2.61	
MAc pH 6.8	0.026 ± 0.028	0.199 ± 0.050	11.43 ± 4.42	*P *<* *0.001
Noradrenaline @ 7 mmHg (Fig. 6D)				
Control pH 7.4	0.227 ± 0.021	0.370 ± 0.028	–6.59 ± 0.44	
MAc pH 6.8	0.078 ± 0.018	0.237 ± 0.040	–6.06 ± 0.44	*P *<* *0.001
Noradrenaline @ 20 mmHg (Fig. 6F)				
Control pH 7.4	0.074 ± 0.012	0.243 ± 0.017	–6.58 ± 0.20	
MAc pH 6.8	0.001 ± 0.017	0.116 ± 0.017	–6.34 ± 0.31	*P *<* *0.001

MAc, metabolic acidosis.

Application of noradrenaline concentration‐dependently decreased the diameter of human thoracic ducts mounted in pressure myographs irrespective of whether the transmural pressure was 7 mmHg (Fig. 6C and D) or 20 mmHg (Fig. 6E and F). Statistical parameters are provided in Table 3.

### Extracellular acidosis inhibits rhythmic contractions of isobarically tested human thoracic ducts mostly at low concentrations of noradrenaline

Human thoracic duct segments investigated under isobaric conditions at pH_o_ 7.4 showed rhythmic oscillatory behavior that waned at high concentrations of noradrenaline (Figs. 6C,E and 7). When the human lymph vessel segments were investigated at pH_o_ 6.8, rhythmic phasic contractions were modest under basal conditions (Fig. 1C and D) but increased substantially in response to noradrenaline: the oscillatory patterns at pH_o_ 6.8 were comparable to those observed at pH_o_ 7.4 when the human thoracic duct segments were exposed to micromolar concentrations of noradrenaline (Fig. 7).

### Depolarization‐induced contraction of isometrically tested human thoracic ducts in response to elevated extracellular [K^+^]

**Figure 8:**
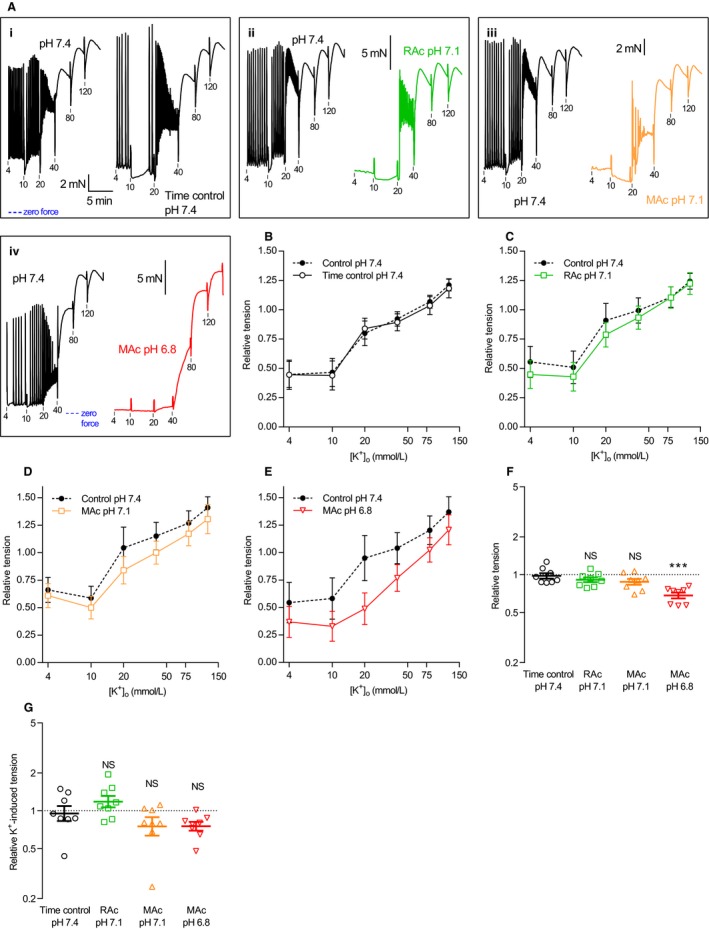
Extracellular acidification has no prominent effect on K^+^‐induced contractions of human thoracic ducts. (A‐E) Original traces (A) and average data (B‐E) showing contractions in response to K^+^‐induced depolarization of human thoracic duct segments mounted under isometric conditions (*n* = 8). The external [K^+^] is indicated on the traces in mmol/L. In panel A, all four sets of traces are plotted on the time scale shown in the first subpanel. In each subpanel, the first trace represents control extracellular conditions of pH 7.4, 5% CO_2_ and 22 mmol/L HCO_3_^–^ whereas the second trace represents the respective interventions: time control (*i*, unchanged acid‐base conditions), respiratory acidosis (*ii*, pH reduced to 7.1 by increasing CO_2_ to 10%) and metabolic acidosis (pH reduced to, *iii*, 7.1 or, *iv*, 6.8 by lowering [HCO_3_^–^] to 11 or 5.5 mmol/L). We performed the time control experiments on matched thoracic duct segments investigated in parallel with – and thus experiencing similar time delays as – the segments exposed to acidosis. The force axes are aligned in each row of panel A and the zero level indicated by the blue horizontal dotted lines. The tension values are expressed relative to that developed during exposure to 120 mmol/L extracellular K^+^ under control conditions (pH 7.4, 5% CO_2_, 22 mmol/L HCO_3_^–^) at the end of each experiment. (F+G) K^+^‐induced cumulative contractile responses (*n* = 8) calculated as the area between the concentration‐response curves and the *x*‐axis (F) or between the curves and the matched baseline value (G) of panel B through E. Note that the data are expressed relative to the matched control curve at pH 7.4, plotted on a log‐scale, and compared by repeated measures one‐way ANOVA followed by Dunnett's posttest. ***P <* *0.001, NS: not significantly different versus time control at pH_o_ 7.4. RAc: respiratory acidosis, MAc: metabolic acidosis.

**Figure 9:**
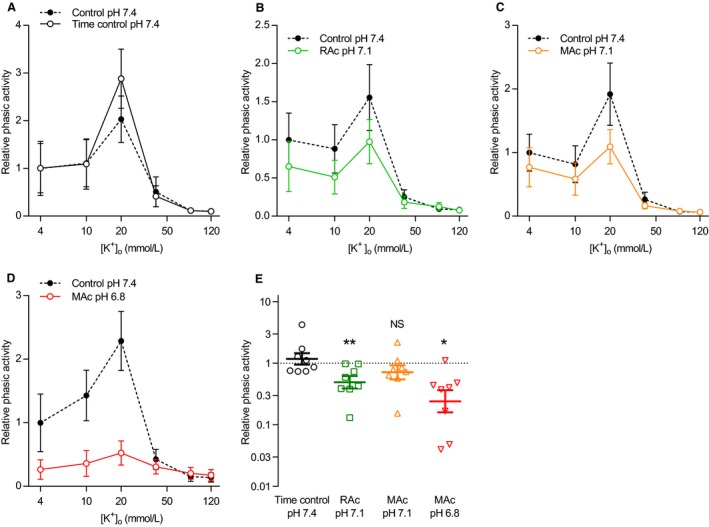
Extracellular [K^+^] and pH modify phasic activity of human thoracic ducts. (A–D) Phasic activity of human thoracic duct segments mounted under isometric conditions (*n* = 8) and exposed to increasing concentrations of K^+^ is expressed relative to the baseline value under control conditions (pH 7.4, 5% CO_2_, 22 mmol/L HCO_3_^–^). Corresponding original force traces are shown in Figure 8A. We performed the time control experiments on matched thoracic duct segments investigated in parallel with – and thus experiencing similar time delays as – the segments exposed to acidosis. The phasic contractile activity – calculated as the average numerical value of the first derivative of the tension curve under each experimental condition – represents a combined measure of amplitude and frequency. (E) Cumulative phasic activity calculated as the area under the curves (*n* = 8) of panel A through D. Note that the data are expressed relative to the matched control curve at pH 7.4, plotted on a log‐scale, and compared by repeated measures one‐way ANOVA followed by Dunnett's posttest. *P <* *0.05, **P <* *0.01, NS: not significantly different versus time control at pH_o_ 7.4. RAc: respiratory acidosis, MAc: metabolic acidosis.

Decreases in pH_o_ below the physiological level of 7.4 inhibit voltage‐gated Ca^2+^‐channels in patch clamp experiments on isolated vascular smooth muscle cells (West et al. 1992; Klockner and Isenberg 1994). Previous reports from arteries also indicate that inhibition of voltage‐gated Ca^2+^‐channels contribute to the decrease in force development during extracellular acidosis (Boedtkjer 2018). We therefore investigated whether extracellular acidification affects force production of human thoracic duct segments in response to depolarization with elevated extracellular [K^+^] (Figs. 8 and 9).

Whereas tension development was reduced in lymphatic vessels contracted at pH_o_ 6.8 compared to pH_o_ 7.4, this was mostly due to the lower tone at baseline: overall tension development was lower at pH_o_ 6.8 (Fig. 8F) but the K^+^‐induced increase in tension from baseline (4 mmol/L K^+^) did not differ significantly among the tested acid‐base conditions (Fig. 8G).

Elevating extracellular [K^+^] to 40 mmol/L or higher almost completely abolished the oscillatory contractile activity of human thoracic ducts – and hence the difference in contractile pattern between human thoracic ducts investigated at normal and low pH_o_ (Fig. 9A–D) – consistent with previous reports that K^+^‐channel activity contributes to contractile rhythmicity of human thoracic ducts (Telinius et al. 2014b).

## Discussion

We show here for the first time that acidosis inhibits the contractile activity of human thoracic ducts. Whereas severe extracellular acidification (pH_o_ reduced to 6.8) is required in order to attenuate overall tension development (Figs. 2 and 3), even moderate acidosis (pH_o_ reduced to 7.1) can under some conditions lower the phasic contractile behavior (Fig. 5). Acid‐base conditions of lymph are highly sensitive to systemic acid‐base disturbances (Witte et al. 1967) and circulatory failure (Berman et al. 1969) and will at least locally reflect interstitial deviations (Palmer et al. 1998). The tested degrees of acidification are well‐within the range of local pH disturbances observed during pathologies, for instance, malignancies (Vaupel et al. 1989; Gerweck and Seetharaman 1996) and ischemia (Mochizuki et al. 1988; Hosseinpour et al. 2014) and even during intense exercise (Street et al. 2001). As the thoracic duct receives mixed lymph from the abdomen and lower extremities, we expect that the pH deviations observed in the human thoracic duct are of smaller magnitude than those observed at the tissue level. Nonetheless, the observation that low pH_o_ inhibits thoracic duct contractions implies that acid‐induced lowering of lymphatic pumping can be a mechanism for edema formation in patients with a wide range of diseases where acid‐base disturbances are part of the pathogenesis or clinical presentation. In addition to accumulation of interstitial fluid, reduced lymphatic pumping activity may also affect metastasis, distribution of pathogens, and immune surveillance.

Notable differences in contractile responses between human thoracic ducts and human lymph vessels of different origins make extrapolations to other parts of the lymphatic network difficult (Sjoberg et al. 1989; Sjoberg and Steen 1991a,b; Telinius et al. 2010). Nonetheless, previous studies on bull mesenteric lymph vessels support that acidosis also relaxes more peripheral lymph vessels (Lobov and Kubyshkina 2001). The contribution of the human thoracic duct to overall lymph drainage is incompletely understood. Contractile activity in the thoracic duct supports lymph flow into the venous blood stream. It also reduces the intravascular pressure in afferent lymph vessel segments and hence the afterload that more peripheral lymph vessels must overcome in order to propel lymph, reduce interstitial fluid build‐up, and prevent edema.

Voltage‐gated Ca^2+^‐channels are required for spontaneous and noradrenaline‐induced contractions of human thoracic ducts in vitro (Telinius et al. 2014c). When compared to arteries (Boedtkjer et al. 2016; Boedtkjer 2018), and considering that acid‐induced inhibition of voltage‐gated Ca^2+^‐channels has been identified in patch clamp experiments on isolated vascular smooth muscle cells (Klockner and Isenberg 1994), it is surprising that contractions of human thoracic ducts are well maintained in response to depolarization with elevated extracellular [K^+^] even during severe extracellular acidification (Fig. 8). Spontaneous contractions of lymph vessels depend on electrical activity that leads to electromechanical activation of smooth muscle cells (Beckett et al. 2007; Imtiaz et al. 2007). The reduced phasic activity of human thoracic ducts at elevated extracellular [K^+^] (Fig. 9) supports a key role for membrane potential control and K^+^‐channel activity in establishing the oscillatory contractile pattern (Telinius et al. 2014b). In light of the well‐recognized capacity of H^+^ to regulate K^+^‐channel activity (Kinoshita and Katusic 1997; Wei and Kontos 1999; Schubert et al. 2001; Dabertrand et al. 2012), our findings point to a likely involvement of K^+^‐channels in acid‐induced inhibition of phasic lymphatic vessel contractions.

The cellular and molecular mechanisms modified by acid‐base disturbances and influencing lymphatic vessel contractions are still unidentified. Because of the chemical equilibria CO_2_ + H_2_O ⇄ H_2_CO_3_ ⇄ HCO_3_^−^ + H^+^, it is not possible to change pH, [HCO_3_^–^], and pCO_2_ independently under normal experimental conditions. Using out‐of‐equilibrium conditions – based on rapid mixing and laminar propulsion of solutions with differing H^+^/HCO_3_^–^/CO_2_ composition – separate signaling consequences of acid‐base equivalents have been identified in resistance arteries (Boedtkjer et al. 2016; Rasmussen and Boedtkjer 2018). The response of resistance arteries to extracellular acid‐base disturbances is complex (Boedtkjer 2018) and relies not only on sensing of extracellular H^+^ but also on separate signaling effects of intracellular H^+^ (Boedtkjer et al. 2011, 2012) and extracellular HCO_3_^–^ (Boedtkjer et al. 2016). It is still controversial whether CO_2_ and intracellular HCO_3_^–^ can have direct vascular effects.

As previously reported, there is substantial inter‐individual and inter‐segment variation particularly in the phasic contractile pattern of lymphatic vessels (McHale and Meharg 1992; Telinius et al. 2010). Despite this variation – which is evident when we compare the individual contractile responses recorded under control conditions of pH 7.4 (Figs. 1A, 2A, 3A, 6A and 8A; summarized for instance in Fig. 1B) – we consistently find that acidosis inhibits human thoracic duct contractions. The reason for the inter‐segment variation is not yet clear but may relate to an uneven distribution of pacemaker cells along the length of the thoracic duct. In our novel isobaric recordings of human thoracic ducts, we more consistently observe spontaneous phasic activity (Fig. 1D) in agreement with the longer, and possibly better coordinated, segments of thoracic duct used for these experiments (Fig. 1C, insert). In congruence with the isometric recordings, the paired design allows us to corroborate the inhibitory effects of acidosis on thoracic duct contractions under isobaric conditions in spite of considerable inter‐individual and inter‐segment variation.

A number of unanswered questions remain for future studies. Beyond the cellular and molecular mechanisms responsible for acidosis‐induced inhibition of lymph vessel contractions and studies of more peripheral human lymph vessels, it remains undetermined whether reduced lymphatic pumping activity influences the risk of metastasis and/or spread of infections. These types of studies will initially depend on development of animal models with modified lymph pumping activity.

In conclusion, we show that contractions of human thoracic ducts are inhibited at low pH_o_. We propose that reduced lymphatic pumping during acidosis can amplify local fluid accumulation caused, for instance, by precapillary dilatation of resistance arteries or increased capillary permeability during ischemia, malignancies or inflammation.

## Conflict of Interest

None declared.
